# Temporal shifts in adaptive immunity drive HIV-associated cryptococcal IRIS: mechanisms, models, and therapeutic opportunities

**DOI:** 10.1515/nipt-2026-0016

**Published:** 2026-07-24

**Authors:** Marcus Hunter, Luis R. Martinez

**Affiliations:** Department of Oral Biology, University of Florida College of Dentistry, Gainesville, FL, USA; Department of Oral Biology, University of Florida College of Dentistry, 1395 Center Drive, DG-48 P.O. Box 100424, Gainesville, FL, 32610, USA; Emerging Pathogens Institute, Gainesville, FL, USA; Center for Immunology and Transplantation, Gainesville, FL, USA; McKnight Brain Institute, Gainesville, FL, USA; Center for Translational Research in Neurodegenerative Disease, University of Florida, Gainesville, FL, USA

**Keywords:** adaptive immunity, *Cryptococcus*, HIV, IRIS, T cells

## Abstract

Cryptococcal meningoencephalitis (CME) remains a leading cause of AIDS-related mortality, and up to one-quarter of persons living with HIV who survive CME develop cryptococcal immune reconstitution inflammatory syndrome (CME-IRIS) after antiretroviral therapy (ART) initiation. CME-IRIS arises when recovering immunity encounters a high residual fungal antigen burden, yet the temporal adaptive immune dynamics that distinguish protective reconstitution from damaging neuroinflammation are not fully defined. This review synthesizes clinical and experimental data to propose a temporal framework centered on CD4^+^ T cell polarization, regulatory failure, memory T cell quality, and T-B cell coordination. Before ART, many patients exhibit a Th2-skewed, low-inflammatory state with impaired Th1 responses, reduced antifungal IgM production, and deficient Tfh-B cell coordination, collectively favoring persistent *Cryptococcus neoformans* antigen burden. With ART, IL-7-driven T cell recovery, Th17/Treg imbalance, Th1/Th17-biased memory responses, and chemokine-guided trafficking of CXCR3^+^CCR5^+^ effector cells into the central nervous system can convert this antigen-rich milieu into fulminant neuroinflammation. We further discuss emerging mouse models that recapitulate unmasking CME-IRIS, the contrasting immune profile of *Cryptococcus gattii*-associated IRIS as a comparative model, and developing therapeutic strategies that include targeted immunomodulators, cell-based therapies, and vaccines. Finally, we highlight future directions – particularly single-cell and spatial transcriptomic profiling of blood and CSF – to resolve which adaptive immune populations drive neuroinflammation and to validate predictive biomarkers for CME-IRIS. A temporally informed view of adaptive immunity in CME-IRIS may refine ART timing, guide risk stratification, and identify new adjunctive interventions for cryptococcosis and related IRIS syndromes.

## Introduction

Cryptococcosis is an invasive fungal infection mainly caused by the encapsulated fungi *Cryptococcus neoformans* and *Cryptococcus gattii* [1]. This life-threatening disease is typically observed in immunocompromised populations such as persons living with HIV (PLWH), with *C. neoformans* being the predominant cause in this group [1]. In PLWH, *C. neoformans* is a major opportunistic pathogen in patients with CD4^+^ T-cell counts below 100 cells/μL, whereas *C. gattii* infection is more common in immunocompetent individuals [1]. The most devastating manifestation of cryptococcosis is cryptococcal meningoencephalitis (CME), the most common fungal disease in AIDS patients [2]. Globally, there are an estimated 152,000 incident cases of HIV-associated CME and 112,000 deaths per year, accounting for approximately 20 % of AIDS-related mortality [2].

The clinical signs of cryptococcosis are often nonspecific and insidious, particularly in PLWH [3]. The most common initial presentation is pulmonary cryptococcosis, reflecting the typical route of acquisition via inhalation of environmental propagules [4]. *C. neoformans* has been associated with exposure to pigeon guano, decaying vegetative matter, and soil [5, 6]. In asymptomatic individuals, pulmonary cryptococcosis is often detected incidentally on chest imaging [7], whereas symptomatic disease can present with fever, chills, cough, malaise, night sweats, dyspnea, weight loss, and hemoptysis [8]. In untreated PLWH, pulmonary cryptococcosis carries a high risk of acute respiratory failure, with rapid clinical progression and substantial mortality [9–12].

**Figure 1:**
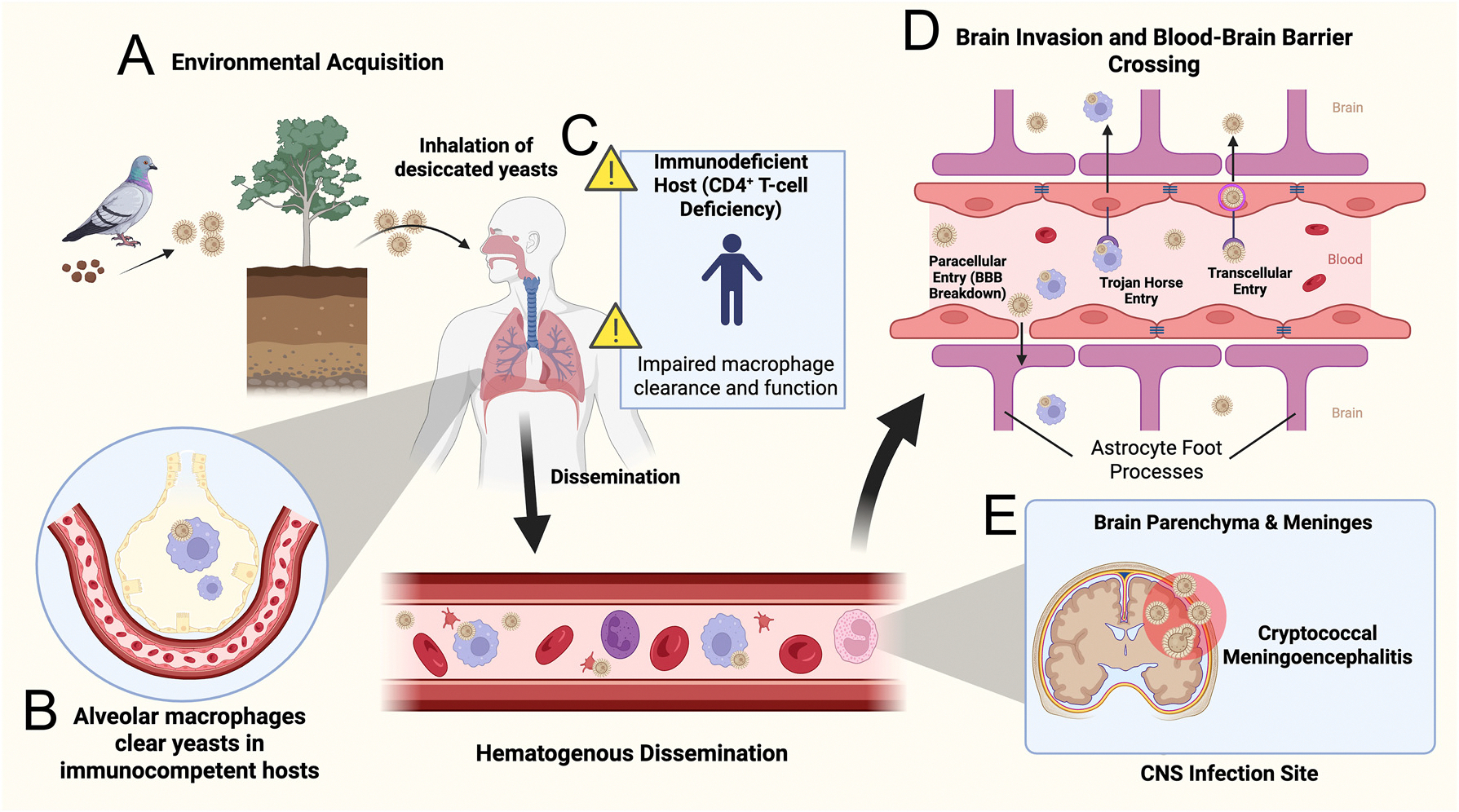
Pathophysiology of cryptococcal meningoencephalitis (CME). (A) *Cryptococcus neoformans* exists in the environment as desiccated yeast cells in pigeon guano and the soil of eucalyptus trees. Following inhalation, (B) yeasts deposit in the alveoli where they are cleared by alveolar macrophages in immunocompetent hosts. (C) Immunodeficient hosts, particularly those with a CD4^+^ T cell deficiency, exhibit impaired macrophage functions resulting in incomplete clearance of the fungi and dissemination through the bloodstream. (D) *C. neoformans* exhibits a high neurotropism where it can cross the blood-brain barrier through any of three different mechanisms: (1) paracellular entry through degradation of tight junctions and passing between the endothelial cells; (2) transcellular entry through CD44-mediated endocytosis and transmigration through endothelial cells; and (3) trojan horse entry whereby infected macrophages cross the blood-brain barrier through either a paracellular or transcellular pathway. (E) Once in the brain, *C. neoformans* establishes in the parenchyma and meninges resulting in CME. The diagram was created with BioRender.com by Marcus Hunter.

The most common and most severe clinical manifestation of cryptococcosis is central nervous system (CNS) infection, usually presenting as CME (Figure 1). Recognized risk factors for *C. neoformans* dissemination and neuroinvasion include HIV with or without AIDS, solid organ transplantation [13], malignancy, cirrhosis, systemic lupus erythematosus [14], and sarcoidosis [15–17]. Symptoms of CME include headache, nausea, vomiting, malaise, altered mental status, and fever [18]. Prominent clinical features are cerebral edema and elevated intracranial pressure (ICP), which can lead to cranial nerve dysfunction, hydrocephalus, seizures, blindness, deafness, and death [18, 19]. Disseminated disease may also involve the skin, prostate, liver, kidneys, bones and joints, eyes, heart, gastrointestinal tract, pancreas, and adrenal glands [20–23].

**Figure 2:**
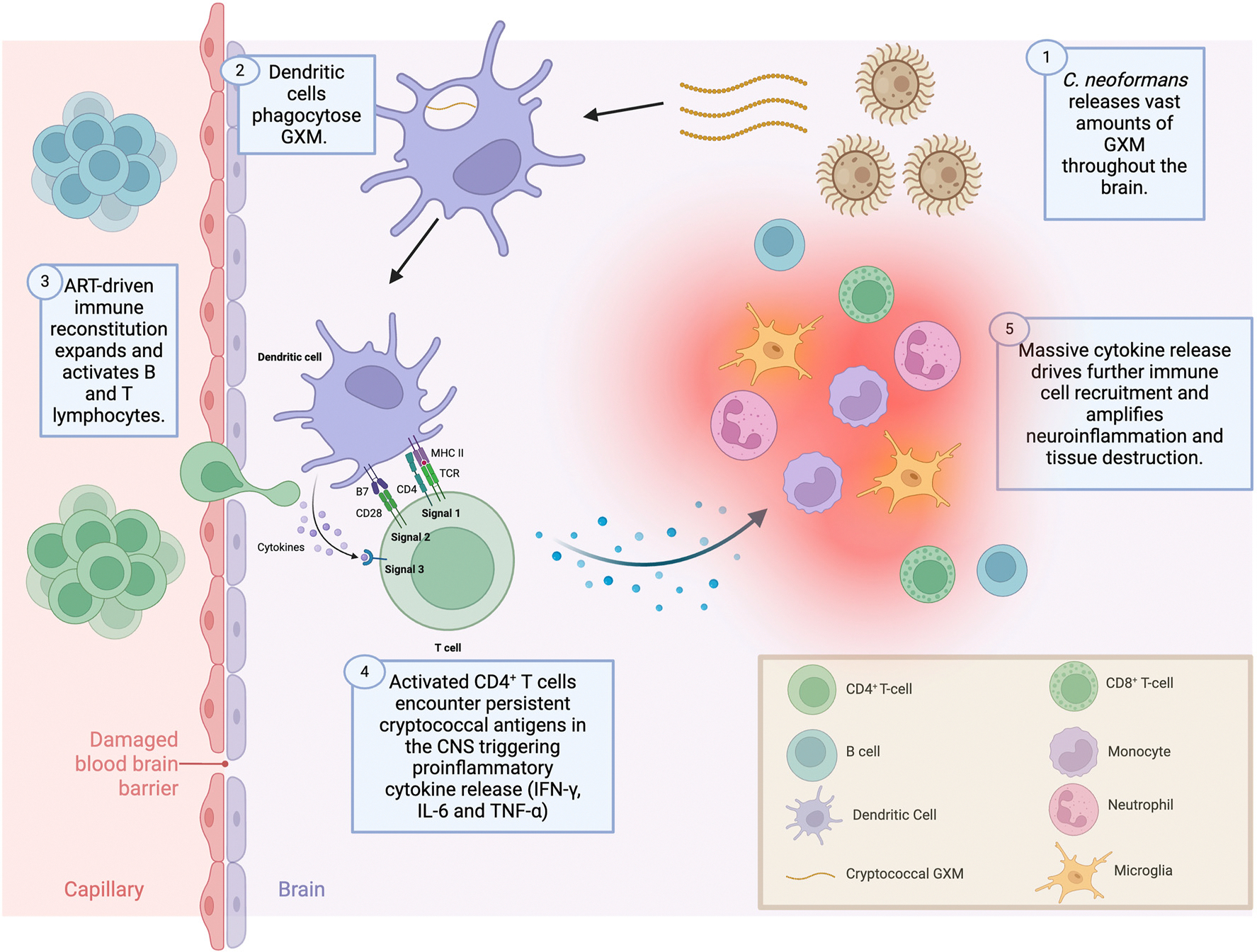
Pathophysiology of CME associated immune reconstitution inflammatory syndrome (CME-IRIS). Incomplete fungal clearance leads to development of CME-IRIS through multiple sequential events: (1) *C. neoformans* releases glucuronoxylomannan (GXM) throughout the brain parenchyma; (2) dendritic cells (and other phagocytic cells) phagocytose the residual GXM; (3) initiation of antiretroviral therapy leads to expansion of B and T lymphocytes; (4) lymphocyte activation occurs as CD4^+^ T cells encounter MHC-II presented GXM, triggering pro-inflammatory cytokine release; and (5) massive cytokine release drives further immune cell recruitment, including CD8^+^ T-cells, B cells, monocytes, microglia and neutrophils, amplifying neuroinflammation and causing further tissue destruction. The diagram was created with BioRender.com by Marcus Hunter.

For PLWH with CME, first-line treatment consists of induction therapy with amphotericin B plus flucytosine for two weeks [24], followed by consolidation therapy with fluconazole 400–800 mg daily for eight weeks [24]. If there is no recurrence of symptoms, maintenance therapy with fluconazole 200 mg daily for at least 12 months is recommended [25]. To reduce the risk of immune reconstitution inflammatory syndrome (IRIS), ART – typically an integrase strand transfer inhibitor combined with two nucleoside or nucleotide reverse transcriptase inhibitors - is initiated four to six weeks after starting antifungal treatment [24, 26]. Cryptococcal IRIS (CME-IRIS) is a pathological condition in which immune recovery paradoxically triggers clinical deterioration due to an excessive inflammatory response to a preexisting cryptococcal infection [27, 28] (Figure 2). Originally categorized as a class 2 pathogen in the damage-response framework, *C. neoformans* has been repositioned as a class 3/4 pathogen following the recognition of CME-IRIS, owing to its capacity to cause extensive damage through either fungal virulence in immunocompromised hosts or IRIS-mediated immunopathology [29, 30]. CME-IRIS typically develops one to two months after CME diagnosis and is associated with early ART initiation (within four weeks of diagnosis) [31–33]. Approximately 25 % of HIV-associated CME patients develop CME-IRIS within the first four months of ART initiation, with an associated mortality of approximately 20 % [34].

CME-IRIS can be classified as unmasking or paradoxical. Unmasking CME-IRIS occurs when a previously unrecognized, asymptomatic cryptococcal infection becomes clinically apparent with new neurological symptoms after ART initiation [35]. In this setting, immune restoration precipitates an inflammatory response to *Cryptococcus* species [32, 36]. Unmasking CME-IRIS typically presents within two to six weeks of ART initiation and occurs in up to 1 % of patients starting ART without a prior diagnosis of cryptococcosis [35, 36]. Paradoxical CME-IRIS is defined as recurrence or worsening of neurological symptoms after initial improvement, often with a negative cerebrospinal fluid (CSF) culture from the index episode, in the context of ART initiation, improved ART adherence, or a change in ART regimen [37]. Paradoxical IRIS generally manifests one to six months after ART initiation [38–40] and occurs in up to 42 % of HIV-associated CME patients, with reported mortality rates of 33–66 % [41]. Risk factors for CME-IRIS include a high initial CSF fungal burden and low baseline inflammatory markers such as CD4^+^ T-cell counts and interferon-gamma (IFN-γ), both of which often increase rapidly after ART initiation [33].

**Figure 3:**
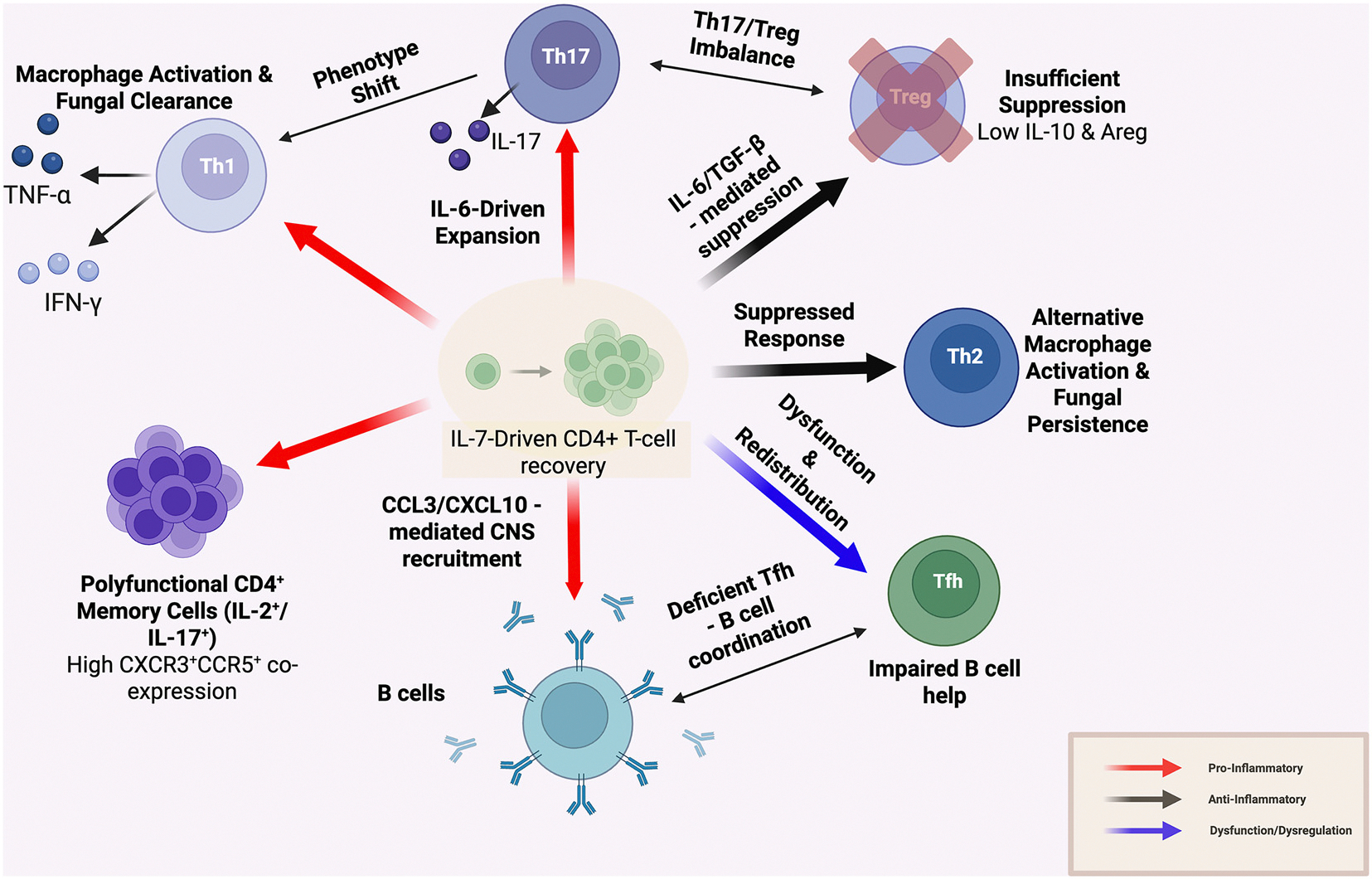
Adaptive immunity at CME-IRIS onset. Following immune reconstitution, multiple pathways contribute to CME-IRIS pathogenesis. Production of Th1 cells and IL-6 driven expansion of Th17 cells contribute to fungal clearance through pro-inflammatory means. The production of polyfunctional memory cells as well as the CNS recruitment of B cells results in a hyperinflammatory CNS environment. In addition, the suppression of Th2 and regulatory T cell responses leads to a repressed anti-inflammatory response. Dysfunctional redistribution of follicular helper T-cells also contributes to CME-IRIS pathogenesis through impaired Tfh-B cell coordination. The diagram was created with BioRender.com by Marcus Hunter.

By 2020, global ART coverage had expanded to approximately 27.5 million adults, up from 15 million in 2014 [2]. Despite this progress, a substantial proportion of individuals – particularly in sub-Saharan Africa – continue to present with advanced HIV disease (CD4^+^ T-cell counts <200/μL) and remain at high risk for opportunistic infections [42]. Increased ART access has therefore enlarged the population at risk for CME-IRIS and other IRIS-related syndromes. Moreover, IRIS is increasingly recognized in other immunosuppressed populations, including solid organ transplant recipients [43] and individuals with multiple sclerosis receiving immune-modulating therapies [44]. This review focuses on the adaptive immune response across the course of ART in PLWH with cryptococcosis and examines how temporal changes in adaptive immunity contribute to the development of CME-IRIS (Figure 3).

## CNS tropism and resident cell interactions

### Regional vulnerability and neuropathology

CME exhibits characteristic patterns of CNS involvement that reflect both the organism’s neurotropism and the host’s immune response. Parenchymal cryptococcal lesions typically manifest in two distinct patterns: superficial lesions affecting the parietal lobe and cerebellum, and deeper lesions concentrated in the basal ganglia [45, 46]. Both patterns commonly present as cryptococcomas – focal masses of yeast cells, inflammatory infiltrate, and gelatinous material that can produce mass effect and contribute to neurological deterioration [45, 46].

Neuroimaging (CT and MRI) studies have documented a diverse spectrum of CNS abnormalities in CME. The most common radiographic findings include meningitis, hydrocephalus, dilation of perivascular spaces, cryptococcomas and lacunar infarction [45, 47, 48]. Additional findings encompass brain atrophy, white matter lesions, brain edema, ventriculitis, encephalitis, mass lesions or nodules, areas of restricted diffusion, granulomas, pseudocysts, cranial nerve enhancement, hemorrhage, vasculitis, calcification and arachnoiditis [47]. Intracranial lesions may develop or progress at multiple anatomical sites throughout the course of the disease, including the periventricular and lateral ventricular regions, basal ganglia, frontal lobe, parietal lobe, occipital lobe, cerebellum, temporal lobe, corona radiata, subcortex, midbrain and centrum semiovale [48, 49].

Neuroimaging findings associated with CME-IRIS demonstrate characteristic features of progressive central nervous system including linear perivascular enhancement within cerebral fissures, choroid plexus enhancement, enhancement of dilated Virchow-Robin space pseudocysts and focal meningeal and parenchymal gadolinium enhancement typically involving the convexities of the cerebral hemispheres [47]. Recognition of these radiographic patterns is critical for timely diagnosis and appropriate management of CME-IRIS.

Histopathological examination of CNS tissue from patients with CME reveals meningitis, meningoencephalitis, encephalitis and ventriculitis as the most dominant patterns of inflammation [50]. The deep gray matter structures are most frequently involved, particularly the basal ganglia (globus pallidus, putamen and caudate nucleus), followed by the midbrain, dentate nucleus, cerebellum, pons and cortex [50]. Importantly granuloma formation – a hallmark of intact cell-mediated immunity – is predominantly observed in non-HIV patients, whereas HIV-infected individuals typically exhibit minimal granulomatous inflammation, reflecting their profound immunodeficiency [50]. This histopathological distinction underscores the central role of adaptive immunity in modulating both tissue damage and fungal containment in CME.

### CNS-resident cell interactions

**Figure 4:**
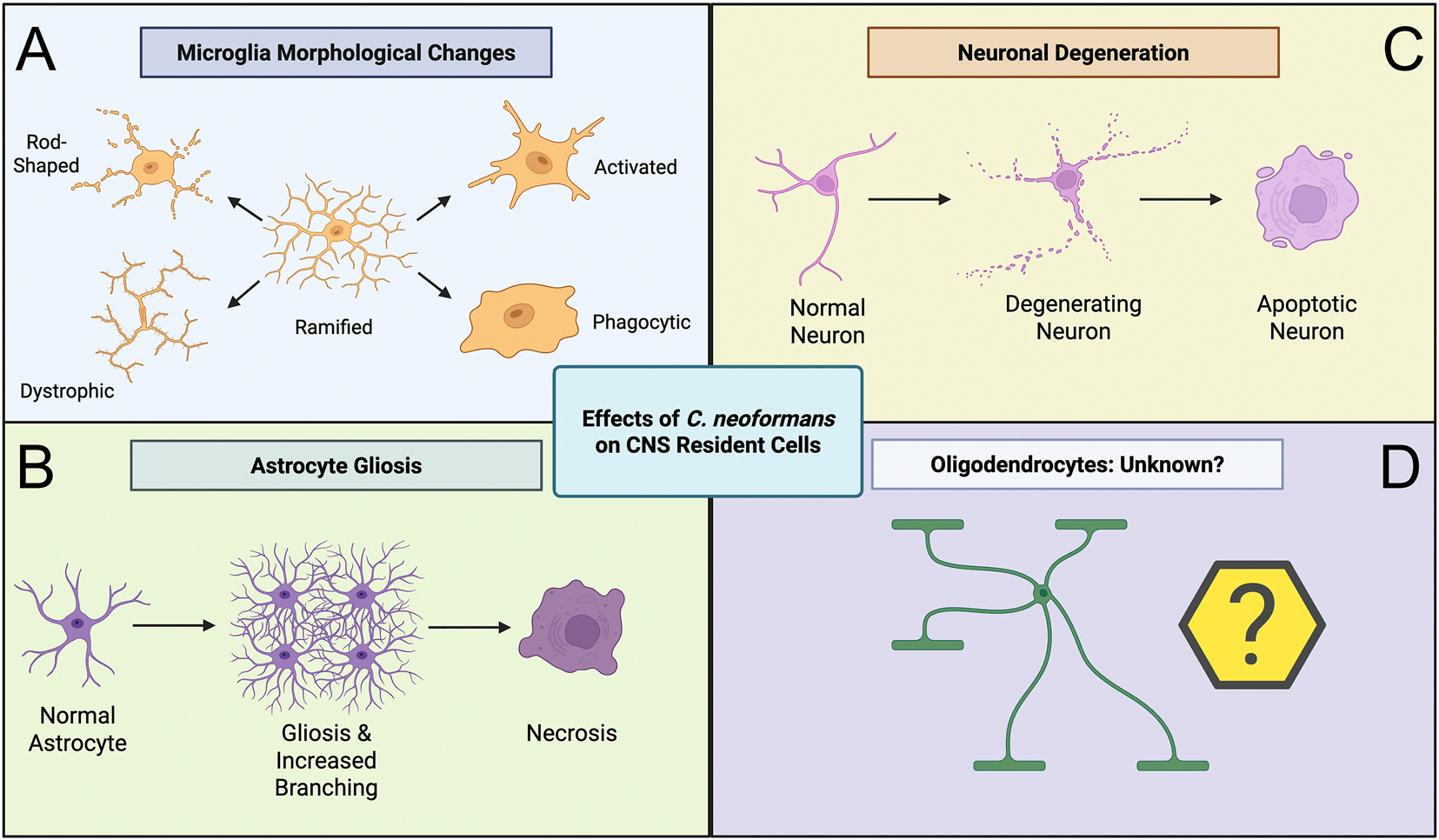
Effects of *C. neoformans* on CNS resident cells. (A) Microglia exhibit five different phenotypes during *C. neoformans* infection including ramified, activated, ameboid/phagocytic, rod-shaped, and dystrophic. (B) Astrocytes exhibit reactive gliosis and increased branching before becoming necrotic in severe cases of CME. (C) Neurons undergo neurodegeneration and apoptosis in response to the *C. neoformans* infection. (D) Oligodendrocytes exhibit transcriptional changes; however further research is needed to understand any structural/functional changes during the course of infection. The diagram was created with BioRender.com by Marcus Hunter.

Beyond the adaptive immune response, CNS-resident cells play critical roles in both host defense against *C. neoformans* and the pathogenesis of tissue injury during CME and CME-IRIS (Figure 4). Microglia, the resident macrophage-like cells of the CNS, serve as the principal reactive cell type during cryptococcal infection, particularly in perivascular and juxtavascular locations [50]. In response to fungal invasion, ramified microglia adopt either an activated amoeboid/phagocytic morphology [50, 51] (Figure 4A); however, microglia near *C. neoformans* cells often exhibit rod-shaped and dystrophic or necrotic features, suggesting that direct fungal-microglial interactions may be cytotoxic or that microglia are overwhelmed by the fungal burden [52]. These observations indicate that while microglia mount an initial response to cryptococcal invasion, their capacity for effective fungal clearance may be limited, particularly in the setting of high organism burden or impaired T-cell help.

Astrocytes, which play essential roles in maintaining the blood-brain barrier, regulating extracellular ion homeostasis, and supporting neuronal function, also undergo pathological changes during CME. Reactive gliosis – a hallmark astrocytic response to CNS injury – is observed in CME (Figure 4B), although it is often limited to areas of large destructive lesions and subpial locations [50, 52]. In severe cases, astrocytes may become necrotized [52]. Transcriptional profiling studies in murine CME models have revealed reduced astrocyte transcriptional signatures, suggesting widespread astrocyte dysfunction or loss during infection [53]. Given astrocytes’ roles in BBB integrity and immune regulation within the CNS, their dysfunction likely contributes to edema formation, inflammation and impaired neuronal support during both acute CME and immune reconstitution.

Neurons are directly affected by cryptococcal infection and the associated inflammatory milieu (Figure 4C). Neurodegeneration and apoptosis have been documented in both human and experimental CME [52, 53]. Transcriptomic analyses demonstrate decreased expression of genes involved in neurotransmission, synaptic connectivity and neuronal structural integrity, indicating broad disruption of neuronal homeostasis [53]. These findings suggest that neurological sequelae in CME survivors may result not only from focal lesions and elevated intracranial pressure but also from diffuse neuronal dysfunction and loss mediated by both direct fungal toxicity and bystander damage from inflammation.

Oligodendrocytes, the myelin-producing cells of the CNS, have received limited attention in the cryptococcal literature but appear to be adversely affected during infection (Figure 4D). Reduced oligodendrocyte transcriptional signatures have been observed in murine models of CME [53], raising the possibility that white matter injury and demyelination contribute to neurological impairment. The extent to which oligodendrocyte damage occurs during CME-IRIS and whether it contributes to long-term cognitive and motor deficits remain important unanswered questions.

Collectively, these findings underscore that CME pathogenesis extends beyond meningeal and perivascular inflammation to encompass widespread dysfunction of CNS-resident cells (Figure 4). Understanding how *C. neoformans* and the host immune response disrupt microglia, astrocytes, neurons and oligodendrocytes is essential for developing neuroprotective strategies that preserve CNS function during antifungal therapy and immune reconstitution. Future studies should also investigate the roles of other issue-resident cells – including ependymal cells, pericytes and CNS-resident lymphocytes – in modulating the balance between fungal clearance and immunopathology in CME and CME-IRIS.

### The polarization response: navigating the Th1/Th2 axis

Type 1 T-helper (Th1) cells and type 2 T-helper (Th2) cells are two of the major subsets of CD4^+^ T helper cells. A Th1-driven host response, characterized by production of IFN-γ and other proinflammatory cytokines, is protective in cryptococcosis [54]; notably, glucuronoxylomannan (GXM), the principal component of the cryptococcal capsule, also drives Th1-mediated immunity [55]. Prior to ART initiation, clinical studies have demonstrated no significant difference in baseline CD4^+^ T-cell counts between HIV-CME patients who subsequently develop IRIS and those who do not [41]. However, pre-ART cytokine profiles demonstrate a paucity of proinflammatory mediators; low serum levels of TNF-α and IFN-γ increase the risk of CME-IRIS [41]. Both TNF-α and IFN-γ enhance fungal clearance through macrophage activation [56]. In parallel, HIV-CME patients at higher risk of developing CME-IRIS display elevated pre-ART plasma levels of Th2 cytokines, including IL-4, IL-5, IL-6, and IL-17 [41, 57, 58]. High IL-4 levels promote alternative activation of macrophages, leading to increased *C. neoformans* proliferation and dissemination [54, 59]. IL-4 can antagonize granulocyte-macrophage colony-stimulating factor signaling and reverse GM-CSF-mediated macrophage killing of cryptococci [60], while IL-6 promotes Th2 differentiation and simultaneously inhibits Th1 polarization [61]. Collectively, a shift toward a Th2-polarized immune environment prior to ART likely results in incomplete fungal clearance, priming an excessive inflammatory response once adaptive immunity is restored upon ART initiation.

After ART initiation, high levels of IL-1Ra, IL-6, and IL-7 are associated with increased risk of IRIS [41]. IL-7 is a major regulator of CD4^+^ T-cell homeostasis, promoting the activation, proliferation, and survival of T cells [62]. Rising IL-7 levels during immune reconstitution drive rapid T-cell regeneration and expansion [63], priming CD4^+^ memory T cells to mount a vigorous response to residual cryptococcal antigens – such as GXM – persisting in the CNS, thereby generating the exaggerated inflammatory response characteristic of IRIS [63]. Understanding how temporal shifts along the Th1/Th2 axis intersect with IL-7-driven T-cell recovery is therefore critical for predicting CME-IRIS risk and identifying immunologic windows for targeted intervention.

### Regulatory failure: the breakdown of the Th17/Treg axis

Th17 cells are defined by their hallmark production of IL-17 [64]. They additionally produce IL-10, IL-21, IL-22, and IL-23, which help sustain inflammatory responses [65, 66]. Notably, Th17 cells exhibit considerable plasticity and can rapidly shift toward a Th1 phenotype [64], and they facilitate neutrophil recruitment, which is critical for the clearance of extracellular pathogens including fungi [67]. Regulatory T cells (Tregs) maintain peripheral immune tolerance through active suppression of immune responses [68]. They express high levels of IL-2Rα and produce inhibitory cytokines including IL-10, TGF-β, and IL-35 [69]. Homeostasis between Th17 cells and Tregs is essential for functional immune balance, and disruption of this equilibrium has been implicated in the pathogenesis of autoimmune diseases [41, 70].

In the context of cryptococcal infection, *C. neoformans* DNA drives expansion of both Th17 cells and Tregs [71]. *C. neoformans* can also inhibit IL-17 expression through production of prostaglandin E2 [72]. Importantly, much of the described Th17 response to *C. neoformans* derives from studies of pulmonary cryptococcosis rather than neurocryptococcosis. Current evidence suggests that Th17 cells play a significant role in pulmonary cryptococcal clearance but may contribute less once bloodstream dissemination has occurred [73]. However, clinical studies indicate that survival in HIV-associated CME correlates with increased IL-17A levels, suggesting a yet-undefined role for Th17 cells in CME pathogenesis [74, 75]. IRIS is also observed in HIV and *Mycobacterium tuberculosis* co-infection, where patients who develop IRIS exhibit increased Th17 levels, further linking Th17 biology to post-ART inflammatory complications [76].

Tregs inhibit excessive Th1 responses through production of IL-10 and amphiregulin (Areg), thereby reducing neuronal damage in patients with HIV-CME [77]. In HIV-positive patients, Treg frequency increases after ART compared with the pre-ART state [78]. In patients with HIV and *M. tuberculosis* co-infection who develop IRIS, lower Treg numbers and high IFN-γ levels are observed following ART [79]. These findings suggest that Tregs play a critical role in suppressing antigen-specific inflammatory responses following immune reconstitution after ART initiation [79].

IL-6 is produced in high amounts after ART initiation and actively promotes Th17 differentiation [80, 81]. In contrast, IL-6 inhibits Treg differentiation, particularly in the presence of TGF-β [70]. Elevated IL-6 levels may therefore perturb the Th17/Treg balance by suppressing Treg-mediated regulation, thereby permitting unchecked proinflammatory responses characteristic of CME-IRIS. Indeed, a Th17/Treg imbalance – accompanied by reduced Treg numbers – is associated with worse clinical outcomes in patients with concomitant AIDS and tuberculosis [76], suggesting an analogous contribution of this disequilibrium to CME-IRIS pathogenesis. Thus, failure of the Th17/Treg axis during immune reconstitution likely represents a key check-point at which dysregulated inflammation converts necessary pathogen control into harmful neuroinflammation, highlighting a potential target for prognostic biomarkers and immunomodulatory therapies in CME-IRIS.

### Memory persistence: tracking memory T cells through reconstitution

Memory T cells mount rapid effector responses upon reencounter with a previously recognized antigen, thereby providing the host with long-term immunological protection [68]. In the pre-ART immune microenvironment, HIV-CME patients who subsequently develop CME-IRIS harbor lower frequencies of CD4^+^ and CD8^+^ memory T cell subsets capable of producing IL-2, IFN-γ, and IL-17 in response to GXM stimulation [42]. After ART initiation, the proportion of CD8^+^ central memory T cells decreases [42]. At CME-IRIS onset, antigenic stimulation elicits a higher proportion of polyfunctional IL-2^+^/IL-17^+^ CD4^+^ T cells [42]; additionally, CD8^+^ effector memory T cells exhibit more robust IL-2 responses to antigenic stimulation in these patients [42]. These findings raise the possibility that IL-2 expression is upregulated to drive expansion of T cells responsive to GXM and other fungal antigens during CME-IRIS, thereby amplifying cytokine production and the exaggerated inflammatory response.

At the time of ART initiation, CD4^+^ and CD8^+^ T cells in CME-IRIS patients also exhibit high co-expression of CXCR3 and CCR5 (CXCR3^+^CCR5^+^) [82]. This CXCR3^+^CCR5^+^ subset represents an effector memory T cell population associated with high IFN-γ production and recruitment to inflamed tissues and is increased in autoimmune diseases such as multiple sclerosis [83]. By analogy, CXCR3^+^CCR5^+^ T cells in CME-IRIS are likely key mediators of neuroinflammation, driving preferential trafficking to the CNS and amplifying local Th1-skewed effector responses [82, 83]. Taken together, these findings highlight that the qualitative features of memory T cell pools and their trafficking programs during ART-driven reconstitution are central determinants of whether immune recovery results in controlled fungal clearance or pathologic neuroinflammatory IRIS.

### Bridging the gap: coordinating the T-B cell axis in CME-IRIS

Follicular helper T (Tfh) cells are characterized by expression of CXCR5, PD-1, ICOS, and IL-21 and normally reside in follicular areas of lymphoid tissue [84]. Tfh differentiation depends on IL-6 and IL-21 [84]. The principal function of Tfh cells is to interact with antigen-primed B cells, promoting their differentiation into immunoglobulin (Ig)-producing plasma cells [84]. Tfh cells have been classified into three main subsets – Tfh1, Tfh2, and Tfh17 – based on their cytokine profiles and B cell help functions [85]. Tfh1 cells secrete IFN-γ and promote IgG2a production; Tfh2 cells secrete IL-4 and drive IgG1 and IgE production; and Tfh17 cells secrete IL-10 and support IgA secretion [85]. To date, no studies have directly examined the role of Tfh cells in cryptococcal infections or CME-IRIS, representing a critical gap in our understanding of the adaptive humoral response in this disease context.

Tfh cells have, however, been extensively studied in HIV infection. Tfh-cell numbers paradoxically increase during HIV infection even as overall CD4^+^ T-cell counts decline, a phenomenon attributed in part to persistent viral replication within lymph node sanctuary sites [86]. This expansion of Tfh cells is accompanied by hypergammaglobulinemia, with elevated serum immunoglobulin levels [87]. In lymph nodes, the number of mononuclear Tfh cells increases post-ART relative to pre-ART [78]. Interestingly, HIV-positive patients who develop IRIS exhibit a lower frequency of Tfh cells compared to those who do not [78]. Furthermore, following ART initiation, certain circulating Tfh subsets display impaired function and reduced survival, and are associated with decreased B-cell receptor diversification [78]. This apparent paradox suggests that in IRIS, overt Tfh hyperactivation may be less central than qualitative dysfunction and spatial redistribution of Tfh cells from lymph nodes into the circulation and peripheral tissues such as the brain, where they may drive localized inflammatory responses.

A subset of HIV-1-infected individuals deemed immunological non-responders exhibits an increased proportion of Tfh cells characterized by CXCR3^+^CCR6^−^ expression [88]. This Tfh subset is associated with IFN-γ-driven inflammation and may promote exaggerated antigen-specific responses characteristic of CME-IRIS or other IRIS manifestations, including those associated with tuberculosis co-infection. Further characterization of Tfh subsets in the lymph nodes and blood of CME-IRIS patients may reveal distinct inflammatory phenotypes – such as CXCR3^+^ Tfh populations – supporting a Tfh-mediated, type 1-skewed humoral and tissue inflammatory mechanism in CME-IRIS. Consequently, dissecting the T-B cell axis in CME-IRIS may uncover novel prognostic markers and identify Tfh-B cell interactions as potential targets for modulating damaging antibody (Ab)– and cytokine-driven neuroinflammation.

### The quiet contributors: the emerging role of B cells in CME-IRIS

In conjunction with T cell-mediated immunity, humoral immunity mediated by B cells is another key component of the adaptive response. Upon antigenic stimulation, B cells proliferate, mature, and differentiate into Ab-secreting plasma cells [89]. They also serve as antigen-presenting cells (APCs), internalizing, processing, and presenting antigens to T cells, and secrete cytokines that shape and sustain immune responses [90]. Together, these functions position B cells as potential contributors not only to fungal control but also to the quality and magnitude of post-ART inflammation in CME-IRIS.

The specific role of B cells in CME-IRIS development remains poorly understood. Patients who develop CME-IRIS exhibit a CSF chemokine profile characterized by increased CCL2 and CCL3/CXCL10 [82]. CCL2 facilitates leukocyte recruitment across the blood-brain barrier (BBB) [91], while the CCL3/CXCL10 axis additionally attracts B cells and other Ab-secreting cells into the CNS [92]. Increased CNS recruitment of B cells – analogous to the T cell trafficking described above – likely contributes to the hyperinflammatory milieu characteristic of CME-IRIS [82, 92], suggesting that B cell CNS trafficking is an underappreciated component of this neuroinflammatory cascade.

In one cohort of HIV-CME patients, those who went on to develop CME-IRIS had lower pre-ART plasma levels of total IgM, laminarin-binding IgM (Lam-IgM), Lam-IgG, and GXM-specific IgM compared with patients who did not develop CME-IRIS [93]. IgM plays a key role in early pulmonary antifungal immunity [94], inhibits Titan cell formation in *C. neoformans* [95], and promotes macrophage-mediated phagocytosis of *C. neoformans* through GXM-specific recognition [96]. Because IgM promotes fungal clearance and limits pulmonary dissemination, reduced IgM levels likely contribute to higher fungal burdens in HIV-CME patients who later develop CME-IRIS [93–96]. Impaired early humoral control of fungal load may therefore set the stage for a more explosive inflammatory response once T cell immunity is restored by ART.

Laminarin is a branched β-(1,3)-glucan found in the *C. neoformans* cell wall and thus represents a relevant Ab target [97]. Abs to these β-glucans have been shown to protect against lethal *C. neoformans* infection in mice [98]. Accordingly, reduced Lam-IgM and Lam-IgG levels may allow higher fungal burdens and increase susceptibility to IRIS in CME patients [93, 97]. These data collectively highlight that both antigen specificity and isotype composition of the antifungal Ab repertoire may modulate the risk of CME-IRIS by influencing fungal clearance before ART.

Concordant with reduced antifungal Ab levels, patients who develop CME-IRIS also exhibit lower pre-ART plasma concentrations of IL-5 and IL-7 [57]. IL-5 is important for driving B cell differentiation, whereas IL-7 supports B cell survival and proliferation [99, 100]. Deficiency of these B cell-supportive cytokines may impair B cell function, limiting effective fungal clearance and creating an antigen-rich environment in which reconstituting CD4^+^ T cells mount an exaggerated inflammatory response [57, 99, 100]. In this scenario, quantitative and qualitative deficits in B cell help and Ab production become early determinants of downstream T cell-driven immunopathology.

Mechanistic studies are needed to clarify how B cells contribute to the pathophysiology of IRIS. There is currently a significant paucity of research specifically examining B cells in CME-IRIS [97]. Further studies are needed to define how B cell subsets and Ab specificities modulate the balance between fungal clearance and inflammation, and how these parameters interact with T cell responses during ART-driven immune reconstitution [97]. Another important dimension is mucosal immunity: lower IgA levels in HIV-positive individuals have been associated with increased risk of CME [97]. Given IgA’s critical role in lung mucosal defense, further research is needed to elucidate how *C. neoformans* evades IgA-mediated immunity to achieve hematogenous dissemination [97, 101]. Additional studies should also interrogate the functionality of B and T cell subsets to identify which populations exert the greatest impact in the inflammatory milieu, thereby informing the design of targeted therapies. Overall, elucidating the role of B cells and humoral immunity in CME-IRIS is essential for building a more complete temporal model of pathogenesis and may reveal novel biomarkers and therapeutic entry points beyond the current T cell–centric paradigm.

### Experimental and clinical perspectives in *Cryptococcus neoformans* CME-IRIS

Dissecting immune evasion and reconstitution mechanisms requires experimental models that recapitulate the clinical pathophysiology of both unmasking and paradoxical IRIS. A mouse model of unmasking CME-IRIS has been developed that reproduces key clinical features including weight loss and increased mortality [102]. This model is established by intranasal infection of Rag1-deficient mice with 100 *C. neoformans* cells three weeks prior to intravenous adoptive transfer of 10^6^ CD4^+^ T cells [102], and is characterized by marked Th1 cell infiltration into the brain with accompanying neuropathological changes including edema, vacuolization, and parenchymal void spaces [102]. Similar Rag1-deficient, CD4^+^ T cell adoptive-transfer strategies have been used to model IRIS-like disease in *Mycobacterium avium* and *Pneumocystis murina* infections, underscoring the generalizability of this approach for studying immune reconstitution-driven pathology [27, 102]. Collectively, these experimental systems provide a tractable platform to interrogate how temporal shifts in T cell–mediated immunity and fungal burden intersect to drive CNS-specific IRIS phenotypes.

A critical gap remains: no mouse model currently faithfully recapitulates the pathophysiology of paradoxical CME-IRIS. Such a model would allow investigators to define mechanisms underlying recurrence or worsening of symptoms despite successful antifungal therapy and to dissect how ART initiation reshapes host–pathogen dynamics over time. These models would be essential for clarifying the coordinated roles of innate and adaptive immune cells, including how evolving T cell subsets, B cells, and myeloid populations converge to produce CNS injury. Moreover, because current models are specifically designed to reflect HIV-associated CME-IRIS, the mechanisms driving IRIS in other immunocompromised populations – such as those receiving immunosuppressive therapies or undergoing solid organ transplantation – may not be adequately captured. Developing complementary models that capture these diverse clinical contexts will be vital for fully understanding IRIS biology and for testing targeted interventions across the spectrum of underlying immune deficits. Ultimately, robust experimental models that mirror the temporal and compartmental complexity of human CME-IRIS are indispensable for translating mechanistic insights into rational timing and design of immunomodulatory therapies.

### Extending the model: *Cryptococcus gattii*-associated IRIS

Although CME-IRIS is classically linked to *C. neoformans*, *C. gattii* can also be associated with IRIS and exhibits a distinct clinical profile [103, 104]. Case reports describe *C. gattii*-associated IRIS in patients with common variable immunodeficiency and hypogammaglobulinemia, implicating a potential protective role for B cells and intact humoral immunity in limiting IRIS risk [103]. *C. gattii* has been proposed to be more immunogenic than *C. neoformans*, as evidenced by higher GXM-IgG levels in HIV-negative CME cases; furthermore, *C. gattii* infections occur predominantly in immunocompetent hosts [97, 103–105]. These observations suggest that host immune status, baseline Ab profiles, and species-specific antigenic features jointly shape the likelihood and phenotype of IRIS. Investigating how *C. gattii* triggers and evades host responses in both immunocompetent and immunocompromised patients will therefore be critical for distinguishing species-specific from shared IRIS mechanisms and for refining antifungal and immunomodulatory strategies that are effective across the *C. neoformans/gattii* species complex. In this way, *C. gattii*-associated IRIS serves as a natural comparator to refine and stress-test temporal immune models derived from *C. neoformans* CME-IRIS.

### Emerging therapeutics for the treatment of CME-IRIS

Beyond improving our mechanistic understanding of CME-IRIS, experimental models enable preclinical evaluation of targeted therapeutic interventions. One emerging concept is that CME-IRIS shares immunopathological features with autoimmune diseases and may therefore be amenable to immunomodulatory interventions. In a mouse model of unmasking CME-IRIS, treatment with star-shaped glatiramer acetate (sGA) reduced mortality and mitigated neuronal cell loss [106]. sGA is an immunomodulatory agent approved for relapsing-remitting multiple sclerosis [107] that suppresses Th1 differentiation. GA-specific helper T cells can cross the BBB and reduce neuroinflammation [108, 109]. Consistent with this mechanism, sGA treatment diminished Th1 and Th17 cells in the unmasking CME-IRIS model [106]. Another avenue involves repurposing targeted anti-inflammatory biologics used in other inflammatory diseases: adalimumab and infliximab (anti-TNF-α) and anakinra (IL-1Rα antagonist) have been used successfully to treat episodes of CME-IRIS and other IRIS manifestations [43, 110–112]. A low-dose IL-2 immune complex that expands regulatory T cells was also shown to ameliorate neurological symptoms and improve survival in mouse models of CME [77]. Drug repurposing offers a relatively rapid and cost-effective strategy to expand the therapeutic armamentarium for CME-IRIS [113]; mouse models provide an essential platform for establishing preclinical proof of concept and optimizing the timing and combinations of such agents. Furthermore, the presence of anti-GM-CSF autoAbs is associated with increased susceptibility to cryptococcosis, supporting an autoimmune contribution to disease pathogenesis [114]. Recombinant cytokines, including GM-CSF or IFN-γ, could thus serve as adjunctive therapies to prevent or treat CME-IRIS, as suggested by studies in which recombinant IFN-γ enhanced resistance to fungal dissemination [115, 116]. Together, these data argue that selectively modulating inflammatory pathways – rather than globally suppressing immunity – may help uncouple fungal control from CNS tissue damage in CME-IRIS.

Ab-based therapies also represent a potential treatment pathway. Production of monoclonal Abs (mAbs) against fungal targets has been shown to enhance host defense in experimental models [117]. For instance, mAbs directed against capsular and cell wall components of *Candida albicans* and *C. neoformans* have demonstrated protective effects in murine infection models, particularly when combined with antifungal drugs [117, 118]. However, the high cost of mAb production and administration poses substantial barriers to widespread use in infectious diseases [118]. Future work must therefore balance the potential benefits of highly specific Ab-based interventions against feasibility and access constraints, particularly in resource-limited settings where the burden of CME-IRIS is greatest.

### A new Frontier: cell-based therapeutics

Another innovative strategy gaining traction is the use of adoptive cell therapies to treat systemic mycoses [119]. NK cell-based adoptive therapies have shown promise in the treatment of malignancies [115]. NK cells can damage fungal hyphae through perforin-mediated cytotoxicity and play an important role in models of neutropenic aspergillosis [120, 121], generating growing interest in NK cell–based adoptive therapies for invasive fungal diseases [115]. T cell-based adoptive therapies are also emerging as promising approaches [119]. One candidate CAR T strategy uses the carbohydrate-recognition domain of the Dectin-1 receptor, which recognizes β-glucans in the fungal cell wall; in an *Aspergillus* conidia model, this Dectin-1-based CAR T cell (D-CAR) inhibited hyphal growth both *in vitro* and *in vivo* [122]. A second CAR design specifically targets GXM polysaccharides within the *C. neoformans* capsule, redirecting CD8^+^ T cells to a major cryptococcal virulence factor [123]. Although considerable research is needed before adoptive cell therapies become clinically viable for invasive fungal diseases – particularly in the context of CME-IRIS – these approaches hold the promise of precisely engineered cellular products capable of enhancing fungal clearance while being temporally calibrated to minimize IRIS-associated immunopathology.

### Preventing CME and CME-IRIS: vaccine candidates

Primary prevention of systemic mycoses and their sequelae, particularly in immunocompromised populations, will ultimately depend on advances in vaccine development. There are currently no licensed vaccines for fungal infections, reflecting major challenges at the fungus-host interface [124]. Fungi can establish clinical latency and lack broadly conserved cross-genus antigens, complicating the design of vaccines with broad coverage [124]. Additionally, most invasive mycoses occur in immunocompromised hosts, complicating efforts to elicit durable memory responses in this patient population [125]. These obstacles underscore the need for vaccine platforms that are both highly immunogenic and specifically tuned to the constraints of immunosuppressed hosts.

Several approaches and advances in vaccine technology demonstrate potential for cryptococcal vaccines. The cryptococcal capsule, composed primarily of GXM and glucuronoxylomannogalactan (GXMGal), is the major virulence factor and remains the most intensively targeted structure in vaccine design [126]. As a result, GXM-conjugate vaccine candidates have shown promise in preclinical models [127, 128]. Semisynthetic vaccine candidates targeting GXM have also been developed, incorporating a synthetic decasaccharide antigen present in serotype A strains responsible for the majority of cryptococcosis cases [129]. Protein-conjugate vaccines are known to induce strong, durable immunity against encapsulated bacteria, and an analogous strategy for *C. neoformans* could, in principle, generate long-lasting resistance to cryptococcal disease [130].

A live-attenuated vaccine has also been developed based on a mutant *C. neoformans* strain lacking a sterylglucosidase (SG) enzyme [131]. Sterylglucosides are glycolipids with immunomodulatory properties [132]. Deletion of the SG enzyme prevents sterylglucoside catabolism, leading to their accumulation and the expression of associated immunomodulatory effects [132]. The *C. neoformans Δsgl1* vaccine candidate is avirulent and protected mice from lethal *C. neoformans* challenge in a CD4^+^ T cell-depleted model [131]. Notably, protection was lost when both CD4^+^ and CD8^+^ T cells were depleted, indicating that SG-mediated protection depends on T cell-driven mechanisms that remain to be fully defined [133]. These findings position SG-based live attenuated strategies as a promising route to engage T cell-mediated antifungal immunity even in the setting of partial CD4^+^ lymphopenia.

Emerging platforms include extracellular vesicle-based vaccines and mRNA vaccines, which could theoretically improve antigen delivery and immunogenicity in immunocompromised hosts [134, 135]. Ultimately, pre- or post-exposure cryptococcal vaccines should aim to generate a robust, antigen-specific Th1 memory response together with protective Ab responses. Such vaccines could reduce fungal burden and thereby diminish the antigen reservoir that fuels CME-IRIS upon ART-mediated immune reconstitution. A candidate capable of eliciting IgM and IgG responses to GXM could additionally compensate for the low baseline GXM-IgM levels observed in CME-IRIS cohorts, potentially reducing both the incidence and severity of IRIS in high-risk individuals.

### Future directions

Current CME-IRIS research has been largely driven by clinical cohort studies comparing HIV-CME patients who do and do not develop IRIS. While these studies have identified numerous correlates and risk factors, the field now urgently requires mechanistic work to advance from association to causation. Building on existing cohort data, single-cell RNA sequencing (scRNA-seq) of patient peripheral blood mononuclear cells and CSF cells could identify which immune cell populations drive the hyperinflammatory milieu of CME-IRIS. scRNA-seq offers the resolution to identify specific T cell, B cell, and Tfh subsets that are quantitatively or qualitatively altered, generating targeted hypotheses for subsequent functional studies. Complementary spatial transcriptomics approaches would further enable investigation of how these subsets interact within brain tissue and CSF compartments, capturing the spatial architecture of neuroinflammation *in situ*. Deeper characterization of cellular interactions and molecular pathways could also refine and validate biomarkers for CME-IRIS. Robust, validated biomarkers would be of major clinical importance for identifying patients at highest risk and implementing timely preventive interventions.

As noted throughout this review, CME-IRIS arises not only in HIV but also in other immunosuppressed conditions, including solid organ transplantation, malignancy, multiple sclerosis, and in individuals receiving various biologic immunosuppressants. While this article has focused on HIV-associated CME-IRIS, comparative mechanistic studies across these clinical settings represent an important next frontier. T and B cell subset dynamics, myeloid responses, and CNS-resident cell behavior may differ substantially depending on the underlying etiology of immunosuppression or the specific agents employed; comparative studies of CME-IRIS in HIV versus non-HIV hosts would therefore yield valuable insights into shared and divergent pathogenic pathways.

Ultimately, cryptococcal IRIS reflects a complex network of interactions spanning multiple immune cell types and anatomical compartments. Although clinical studies have substantially expanded our understanding of associations between cell subsets, cytokine profiles, and outcomes, much more work is needed to dissect the underlying mechanisms that drive IRIS. A more mechanistic, temporally resolved understanding of these pathways will not only enable better-targeted therapies for CME-IRIS but may also identify general principles and interventions applicable to other forms of IRIS across diverse infectious and noninfectious contexts.

## Data Availability

Not applicable.
